# Percutaneous coronary intervention in patients undergoing transcatheter aortic valve implantation: a systematic review and meta-analysis

**DOI:** 10.1007/s12471-023-01824-w

**Published:** 2023-11-01

**Authors:** Hugo M. Aarts, Nicole D. van Hemert, Timion A. Meijs, Astrid C. van Nieuwkerk, Jurriën M. ten Berg, Joanna J. Wykrzykowska, Niels van Royen, Carl E. Schotborgh, Pim A. L. Tonino, Alexander IJsselmuiden, Tessel N. Vossenberg, Gert K. van Houwelingen, Ton Slagboom, Michiel Voskuil, Ronak Delewi

**Affiliations:** 1 Department of Cardiology, University Medical Centre Utrecht, Utrecht, The Netherlands; 2 Department of Cardiology, Amsterdam University Medical Centre, Amsterdam, The Netherlands; 3 Department of Cardiology, St. Antonius Hospital, Nieuwegein, The Netherlands; 4 Department of Cardiology, Maastricht University Medical Centre, Maastricht, The Netherlands; 5 Department of Cardiology, Groningen University Medical Centre, University of Groningen, Groningen, The Netherlands; 6 Department of Cardiology, Radboud University Medical Centre, Nijmegen, The Netherlands; 7 Department of Cardiology, Haga Hospital, The Hague, The Netherlands; 8 Department of Cardiology, Catharina Hospital, Eindhoven, The Netherlands; 9 Department of Cardiology, Amphia Hospital, Breda, The Netherlands; 10 Department of Cardiology, Medical Centre Leeuwarden, Leeuwarden, The Netherlands; 11 Department of Cardiology, Thorax Centre Twente, Medical Spectrum Twente, Enschede, The Netherlands; 12 Department of Cardiology, Onze Lieve Vrouwe Gasthuis, Amsterdam, The Netherlands

**Keywords:** Aortic Valve Stenosis, Transcatheter Aortic Valve Implantation, Coronary Artery Disease, Percutaneous Coronary Intervention

## Abstract

**Objective:**

The importance of revascularisation of significant coronary artery disease (CAD) in patients undergoing transcatheter aortic valve implantation (TAVI) is unclear. Despite the lack of randomised controlled trials comparing different revascularisation strategies, guidelines currently recommend percutaneous coronary intervention (PCI) in patients with significant proximal CAD undergoing TAVI.

**Methods:**

In this systematic review and meta-analysis, a systematic search was conducted to identify studies comparing TAVI with and without PCI in patients with significant CAD on pre-TAVI coronary angiography. Endpoints were all-cause mortality, cardiac death, stroke, myocardial infarction and major bleeding.

**Results:**

In total, 14 studies were included, involving 3838 patients, of whom 1806 (47%) underwent PCI before TAVI. All-cause mortality did not differ significantly between TAVI with and without preceding PCI at 30 days, 1 year and > 1 year. There were no significant differences in risk of cardiac death, stroke or myocardial infarction between the groups. However, TAVI performed with PCI resulted in a higher risk of major bleeding within 30 days after TAVI (odds ratio: 0.66; 95% confidence interval: 0.46–0.94).

**Conclusion:**

This systematic review and meta-analysis showed no significant differences in clinical outcomes between patients with concomitant significant CAD who were treated with TAVI with and without preceding PCI at both short- and long-term follow-up. However, there was a higher risk of major bleeding at 30 days in patients undergoing TAVI with preceding PCI. In the context of serious risk of bias in the included studies, results of randomised controlled trials are warranted.

**Supplementary Information:**

The online version of this article (10.1007/s12471-023-01824-w) contains supplementary material, which is available to authorized users.

## What’s new?

- This is the first systematic review and meta-analysis comparing the clinical outcomes of patients with concomitant significant coronary artery disease undergoing transcatheter aortic valve implantation (TAVI) with or without preceding percutaneous coronary intervention (PCI).
- Patients undergoing TAVI without PCI had similar clinical outcomes, including mortality, at both short- and long-term follow-up as those treated with TAVI with PCI.
- Patients undergoing TAVI with PCI had a higher risk of major bleeding.
- In the context of serious risk of bias, results from well-organized randomised controlled trials, such as the ongoing PRO-TAVI and NOTION-3 trials, are warranted.

## Introduction

Transcatheter aortic valve implantation (TAVI) is a safe treatment modality for patients with symptomatic severe aortic valve stenosis, irrespective of their surgical risk profile [1, 2]. The prevalence of coronary artery disease (CAD) in patients undergoing TAVI is reported to be 40% to 75% [3]. Although international guidelines recommend treatment of coexisting CAD in patients undergoing surgical aortic valve replacement, the importance of coronary revascularisation prior to TAVI is unclear [4–6]. Small observational studies have failed to show a beneficial effect of revascularisation of significant CAD on clinical outcomes in patients undergoing TAVI. Nevertheless, international guidelines recommend considering percutaneous coronary intervention (PCI) for stenoses ≥ 70% in proximal segments in patients scheduled to undergo TAVI [5, 6].

However, PCI is not without risk in patients with severe aortic valve stenosis. First, patients undergoing TAVI are characterised by high age, which is often accompanied by highly calcified coronary arteries, thereby increasing the risk of periprocedural complications during PCI. The presence of severe aortic valve stenosis limits the ability to compensate for these life-threatening periprocedural complications. Furthermore, PCI before TAVI necessitates the use of dual antiplatelet therapy (DAPT) during the TAVI procedure. On the other hand, complete revascularisation of significant CAD can theoretically decrease the risk of myocardial ischaemia during rapid pacing. Moreover, coronary access after TAVI may be technically challenging due to the presence of the TAVI prosthesis. Currently, debate among interventional cardiologists has led to different revascularisation strategies in patients with concomitant significant CAD undergoing TAVI. Overall, well-organised, large-scale studies comparing TAVI with or without preceding PCI are lacking.

This is the first systematic review and meta-analysis comparing the clinical outcomes of patients with concomitant significant CAD undergoing TAVI with or without preceding PCI.

## Methods

This systematic review and meta-analysis were performed in accordance with the Preferred Reporting Items for Systematic Reviews and Meta-analyses (PRISMA) guidelines. On 20 July 2022, a systematic search was conducted in PubMed, Embase, Cochrane Library and the database of the National Health Service Centre for Reviews and Dissemination. Additionally, reference lists from the included studies and relevant reviews were checked for additional eligible studies. The full search strategy is shown in Table S1 in the Electronic Supplementary Material.

### Eligibility criteria

Studies were included in this systematic review and meta-analysis if the following criteria were met: (1) patients with severe aortic valve stenosis were treated with TAVI; (2) patients had concomitant significant CAD as defined by local guidelines on pre-TAVI coronary angiography; (3) treatment of significant CAD consisted of medical therapy only (TAVI only group) or PCI before TAVI (TAVI + PCI group); (4) a comparison was made between the 2 treatment groups, and clinical outcomes were reported at set time intervals; and (5) the publication was written in English. Ongoing trials, case reports and reviews were excluded.

Trials with > 2 arms for which a subset of interventions fulfilled the inclusion criteria were kept in the meta-analysis after discarding the arms that did not fulfil the inclusion criteria. In case of missing event rates, the authors of the publication were contacted to request additional information. Subsequently, studies were included in the systematic review and meta-analysis if absolute event rates were provided.

### Study selection

Two reviewers (HMA and NDH) independently screened studies for eligibility based on the title and abstract. If one reviewer deemed a study relevant for inclusion, the full text was assessed for eligibility. Subsequently, full texts of potentially eligible studies were assessed by both reviewers. In case of disagreement, consensus was reached by consulting a third reviewer (RD).

### Data extraction and endpoints

Predefined data extraction included name of first author, year of publication, study design, inclusion period, sample size, definition of significant CAD, TAVI access, and TAVI prosthesis. Endpoints were all-cause mortality, cardiac death, stroke, myocardial infarction (MI) and major bleeding after TAVI. To determine the odds ratio (OR) for each study, absolute event rates were extracted. If relative event rates were reported, the reviewers calculated the absolute numbers by using the reported sample size. Endpoints were assessed at 30 days, 1 year and > 1 year following TAVI.

### Quality assessment

The certainty of evidence and risk of bias for all included studies were assessed independently by 2 reviewers (HMA and NDH) using the Grading of Recommendations, Assessment, Development and Evaluation (GRADE) approach [7]. Confounding factors were specified prior to risk of bias assessment and included risk scores for both periprocedural mortality and complexity of CAD.

### Data synthesis and analysis

The DerSimonian and Laird random-effects model was used to calculate the pooled OR with 95% confidence interval (CI) for all endpoints. OR > 1 indicates an increased risk for patients undergoing TAVI only. In case data were insufficient to determine OR, a narrative synthesis was reported. Statistical heterogeneity between included studies was calculated using the Cochran Q statistic (I^2^), with I^2^ scores > 60% indicating substantial heterogeneity. Moreover, as the definition of significant CAD varied between studies, sensitivity analyses were performed on studies with a cut-off value of 50% for significant coronary artery stenosis and studies with a cut-off value of 70%.

To identify potential publication bias, a funnel plot was created for every outcome and time interval, and potential missing studies were detected and adjusted by the trim-and-fill method. The funnel plots were inspected visually and quantified on asymmetry using the Egger test.

Two-tailed *p*-values of < 0.05 were considered to be statistically significant. All analyses were performed using Comprehensive Meta-Analysis version 3 (Biostat Inc., Englewood, NJ, USA).

## Results

### Systematic search

The systematic search yielded 1640 studies. A total of 14 studies—13 observational studies and 1 randomised controlled trial (RCT)—were included in this meta-analysis (see Figure S1 in Electronic Supplementary Material), with a total of 3838 patients [8–21]. Importantly, different definitions for significant CAD were used, with cut-off values for significant lesions ranging from 50% to 75% obstruction in a major coronary artery.

A total of 1806 patients (47%) underwent TAVI with preceding PCI, whereas 2032 patients (53%) with significant CAD underwent TAVI only. Two studies included patients who underwent PCI either before TAVI or concomitantly with TAVI [11, 12]. Haemodynamic parameters (e.g. fractional flow reserve) were used in 2 studies [16, 20], and 2 studies assessed the complexity of CAD by using the SYNTAX score [11, 19]. The majority of patients (86.7%) underwent transfemoral TAVI. A balloon-expandable TAVI device was implanted in 59.6% of the patients. Characteristics and event rates of included studies are presented in Table 1 and Tables S2–S4 in the Electronic Supplementary Material.

**Table 1:** Characteristics of included studies in systematic review and meta-analysis

First author name, publication year (reference)	Study design	Inclusion period	Total cohort size (*N*)	CAD definition	CAD patients (*n*)	PCI in CAD (*n*)	Risk score^a^	LVEF, %^a^	TF-TAVI	TAVI valve
Barbanti et al., 2017 [12]	Prospective registry	2013–2017	604	≥ 70% stenosis in major epicardial artery (or ≥ 50% if LM or vein graft)	134 (23%)	51 (39%)	PCI STS 3.7 (2.1–5.4) No PCI STS 3.8 (2.8–5.6)	PCI 55 (45–60) No PCI 55 (45–60)	99%	BE valve: 32% SE valve: 68%
Zivelonghi et al., 2017 [8]	Retrospective registry	2010–2016	287	≥ 50% stenosis	123 (43%)	34 (28%)	PCI ES 32.7 ± 22.4 No PCI ES 36.0 ± 24.8	Unknown	80%	BE valve: 79%
Elyasi et al., 2018 [14] (Abstract)	Retrospective registry	NA	474	Unknown	165 (35%)	92 (56%)	Unknown	Unknown	Unknown	Unknown
Huczek et al., 2018 [19]	Retrospective registry	2009–2015	896	> 70% stenosis in epicardial coronary vessel > 1.5 mm (> 50% for LM)	462 (52%)	169 (37%)	Unknown	PCI 52.4 ± 12.7 No PCI 52.7 ± 12.3	83%	SE valve: 65%
Millan-Iturbe et al., 2018 [15]	Prospective registry	2007–2016	944	≥ 70% stenosis or ≥ 50% in LM	244 (26%)	136 (56%)	PCI STS 5.5 ± 3,4 No PCI STS 5.3 ± 2.7	Unknown	94%	BE valve: 8% SE valve: 71%
Cazé et al., 2019 [18] (Abstract)	Retrospective registry	2014–2017	526	Unknown	203 (39%)	109 (54%)	Unknown	Unknown	Unknown	Unknown
Elbaz et al., 2020 [21]	Retrospective registry	2012–2017	1967	> 70% stenosis in any LAD, RCX or RCA (or > 50% stenosis in LM)	888 (45%)	444 (50%)	Unknown	Unknown	85%	BE valve: 41%
Young et al., 2020 [17] (Abstract)	Retrospective registry	2012–2018	2729	> 50% stenosis in unprotected LM (or > 70% in proximal LAD)	160 (6%)^b^	102 (64%)	Unknown	Unknown	Unknown	Unknown
Boogert et al., 2021 [20]	Cohort study	2007–2018	1323	> 50% stenosis	577 (44%)	150 (26%)	PCI STS 4.7 (3.3–6.5) No PCI STS 4.3 (3.0–6.2) PCI ESII 3.9 (2.6–7.2) No PCI ESII 3.58 (2.3–5.7)	Unknown	71%	BE valve: 80%
Dagan et al., 2021 [13]	Prospective registry	2008–2018	324	≥ 50% stenosis in ≥ 1 of major coronary vessels	137 (42%)	48 (35%)	Unknown	Unknown	96%	BE valve: 28% SE valve: 72%
Duran Karaduman et al., 2021 [11]	Retrospective registry	2011–2019	526	> 70% stenosis in epicardial coronary vessel > 1.5 mm (or > 50% stenosis for LM)	127 (24%)	65 (51%)	PCI ESII 7.4 (4.7–11.2) No PCI ESII 8.6 (5.2–13.2)	PCI 55.9 (45.0–63.5) No PCI 55.0 (40.0–65.0)	Unknown	BE valve: 94%
Kaihara et al., 2021 [10]	Retrospective registry	2016–2018	186	> 75% stenosis in ≥ 1 major branch (or 50% stenosis only in LM on CAG/CTA)	78 (42%)	32 (41%)	Unknown	Unknown	Unknown	BE valve: 87% SE valve: 13%
Matta et al., 2021 [9]	Retrospective registry	2016–2020	1030	≥ 50% stenosis in major coronary vessel	372 (36%)	255 (69%)	PCI STS 6.6 ± 4.9 No PCI STS 5.8 ± 4.3 PCI ES 14.1 ± 10.2 No PCI ES 14.6 ± 9.2	PCI 53.9 ± 11.6 No PCI 53.4 ± 12.1	94%	BE valve: 58%
Patterson et al., 2021 [16]	RCT	2012–2017	Unknown	≥ 70% stenosis in major epicardial artery (or ≥ 50% if protected LM or vein graft)	235 (unknown)	119 (51%)	PCI STS 4.4 (1.3–26.9) No PCI STS 4.4 (1.1–36.5) PCI ES 11.1 (1.4–63.8) No PCI ES 13.9 (1.2–77.4)	Unknown	78%	BE valve: 84%

*BE* balloon-expandable, *CAD* coronary artery disease, *CAG* coronary angiography, *CTA* computed tomography angiography, *ES(II)* EuroSCORE (II), *LVEF* left ventricular ejection fraction, *NA* not applicable, *PCI* percutaneous coronary intervention, *RCA* right coronary artery, *RCX* ramus circumflex coronary artery, *RCT* randomised controlled trial, *SE* self-expandable, *STS* Society of Thoracic Surgeons score, *TAVI* transcatheter aortic valve implantation, *TF* transfemoral

^a^ Data are median (interquartile range) or mean ± standard deviation

^b^ Left main coronary artery (*LM*) and proximal left anterior descending coronary artery (*LAD*) lesions only

### Certainty of evidence

Quality assessment of individual studies revealed serious risk of bias in all observational studies (Table S5 in Electronic Supplementary Material). This was primarily due to the absence of a standardised protocol for the decision on performing PCI prior to TAVI, unclear criteria for outcome ascertainment and/or absence of adjustment for important confounders. Visual assessment of funnel plots raised some concern for publication bias for several endpoints, but this was not confirmed by the Egger test in any case (see Figure S2 in Electronic Supplementary Material). An overview of the quality assessment and certainty of evidence as assessed by the GRADE approach is presented in Tables S5–S7 in the Electronic Supplementary Material.

### All-cause mortality

**Fig. 1:**
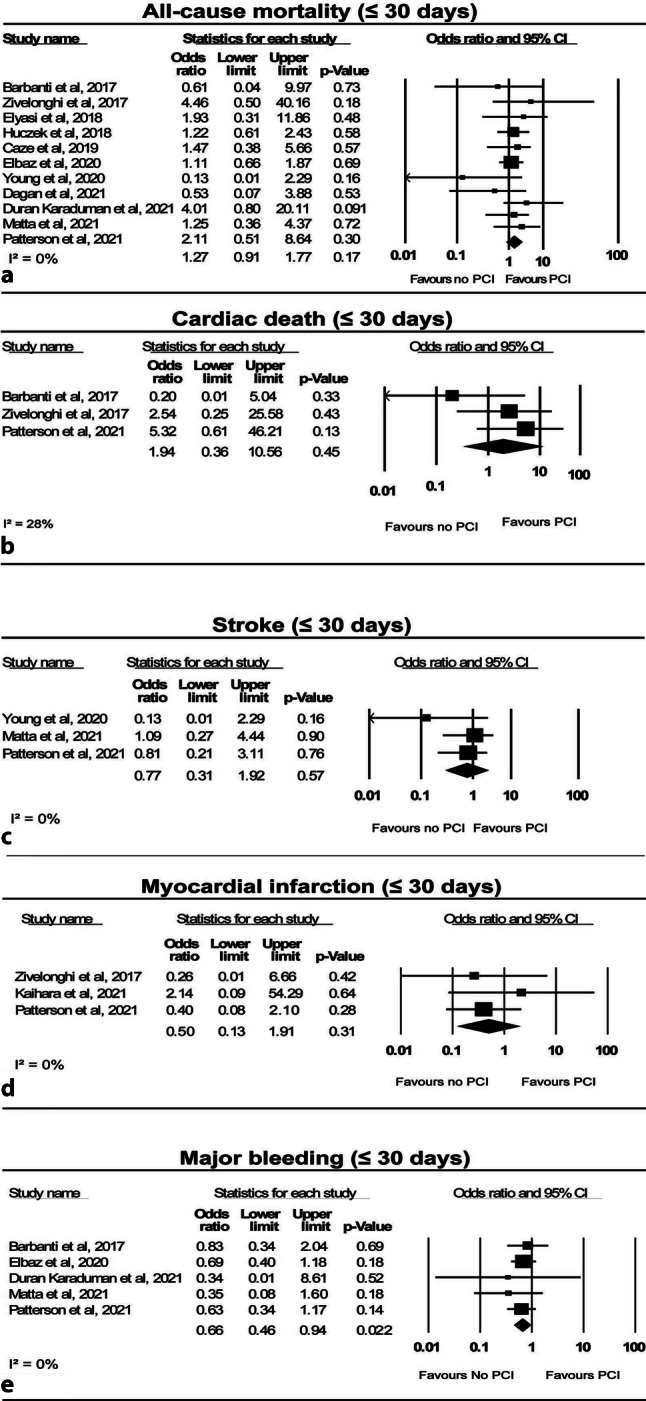
Forest plots for short-term clinical outcomes, **a** all-cause mortality, **b** cardiac death, **c** stroke, **d** myocardial infarction and **e** major bleeding. *PCI* percutaneous coronary intervention

**Fig. 2:**
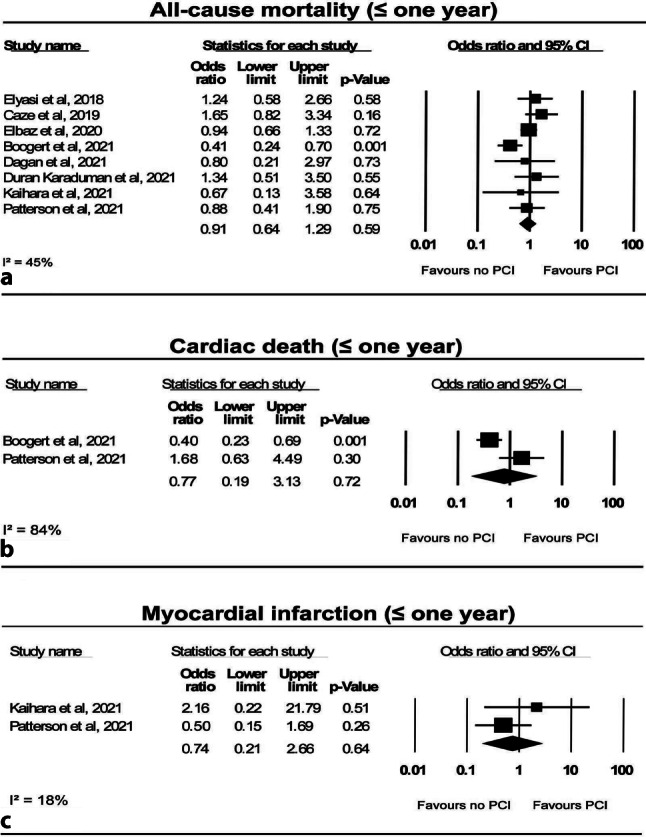
Forest plots for mid-term clinical outcomes, **a** all-cause mortality, **b** cardiac death and **c** myocardial infarction. *PCI* percutaneous coronary intervention

**Fig. 3:**
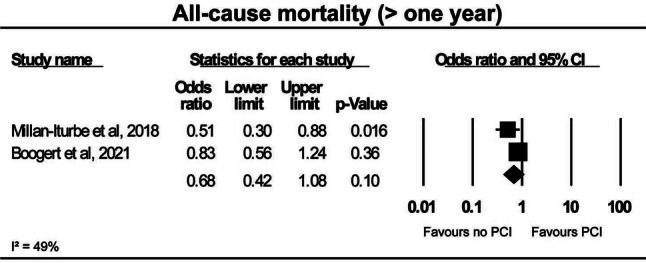
Forest plot for all-cause mortality > 1 year. *PCI* percutaneous coronary intervention

All-cause mortality was similar in patients undergoing TAVI only and patients undergoing TAVI and PCI at 30 days (5.9% vs 4.7%; OR: 1.27; 95% CI: 0.91–1.77; *p* = 0.17; I^2^: 0%) (Fig. 1a; [8, 9, 11–14, 16–19, 21]) and 1 year (13.6% vs 16.4%; OR: 0.91; 95% CI: 0.64–1.29; *p* = 0.59; I^2^: 45%) (Fig. 2a; [10, 11, 13, 14, 16, 18, 20, 21]). Two studies reported on all-cause mortality > 1 year, but no significant difference was found between patients with TAVI only and those with TAVI and PCI (31.5% vs 67.7%; OR: 0.68; 95% CI: 0.42–1.08; *p* = 0.10; I^2^: 49%) (Fig. 3a; [15, 20]).

### Cardiac death

Cardiac death rates did not differ between patients with TAVI only and those with PCI before TAVI at 30 days (3.3% vs 1.5%; OR: 1.94; 95% CI: 0.36–10.56; *p* = 0.45; I^2^: 28%) (Fig. 1b; [8, 12, 16]) and 1‑year follow-up (8.1% vs 12.3%; OR: 0.77; 95% CI: 0.19–3.13; *p* = 0.72; I^2^: 84%) (Fig. 2b; [16, 20]).

### Stroke

The stroke incidence at 30 days was similar between patients treated with TAVI only and those undergoing TAVI with preceding PCI (1.3% vs 2.6%; OR: 0.77; 95% CI: 0.31–1.92; *p* = 0.57; I^2^: 0%) (Fig. 1c; [9, 16, 17]). One-year stroke incidence was 4.9% and 4.6% for patients undergoing TAVI only and patients TAVI with preceding PCI, respectively [10, 16].

### Myocardial infarction

MI rates were similar in patients treated with TAVI only and patients undergoing TAVI and PCI at 30 days (1.1% vs 2.1%; OR: 0.50; 95% CI: 0.13–1.91; *p* = 0.31; I^2^: 0%) (Fig. 1d; [8, 10, 16]), and they remained similar at 1‑year follow-up (4.3% vs 6.0%; OR: 0.74; 95% CI: 0.21–2.66; *p* = 0.64; I^2^: 18%) (Fig. 2c; [10, 16]). Neither type of MI nor subsequent treatment was reported.

### Major bleeding

Patients treated with TAVI only had a significantly lower risk of major bleeding during the first 30 days (7.4% vs 9.4%; OR: 0.66; 95% CI: 0.46–0.94; *p* = 0.022; I^2^: 0%) (Fig. 1e; [9, 11, 12, 16, 21]). Major bleeding at 1 year was assessed in 1 study, which reported an incidence of 18.1% in patients undergoing TAVI only versus 26.1% in those with PCI before TAVI (*p* = 0.19) [16].

### Sensitivity analysis

Eight studies defined significant CAD as a stenosis ≥ 70% in a major epicardial coronary artery [10–12, 15–17, 19, 21]. In line with the results of the meta-analysis on all studies, sensitivity analysis showed a significantly lower risk of major bleeding in patients treated with TAVI only compared with those undergoing TAVI and PCI (OR: 0.68; 95% CI: 0.47–0.99; *p* = 0.043; I^2^: 0%) (Figure S3 in Electronic Supplementary Material). No differences between the 2 groups were found in other clinical outcomes (Figure S3 in Electronic Supplementary Material).

Four studies used a cut-off value of 50% in their definition of significant CAD [8, 9, 13, 20]. The sensitivity analysis showed no significant difference in all-cause mortality within 30 days. However, TAVI without preceding PCI resulted in a significantly lower incidence of all-cause mortality at 1 year (OR 0.45; 95% CI: 0.28–0.74; *p* = 0.002; I^2^: 0%) (Figure S4 in Electronic Supplementary Material). No data on other clinical outcomes were available in these studies.

Moreover, the results of the meta-analysis using the random-effects model persisted in the fixed-effect models.

## Discussion

The main conclusions of this systematic review and meta-analysis were: (1) TAVI without preceding PCI for concomitant significant CAD was associated with similar clinical outcomes, including mortality, compared with TAVI with PCI; (2) patients undergoing TAVI with PCI were more likely to suffer from major bleeding during the first 30 days following TAVI; and (3) in studies using a cut-off value for significant coronary artery stenosis of 50%, TAVI without preceding PCI resulted in a lower risk of all-cause mortality during the first year.

This is the first systematic review and meta-analysis comparing clinical outcomes of patients with concomitant significant CAD undergoing TAVI with or without preceding PCI. Severe CAD as indicated by high SYNTAX scores has been shown to be associated with poorer clinical outcomes following TAVI [22, 23]. Based on these findings, PCI is often performed in patients with significant CAD undergoing TAVI. However, our results indicated that TAVI without preceding PCI in patients with concomitant significant CAD undergoing TAVI yields comparable results to TAVI combined with PCI at both short- and long-term follow-up. Although these findings are in line with the results of previous studies [24, 25], these studies defined CAD different than we did as they included patients without concomitant significant CAD at the time of TAVI but with a history of prior revascularisation or MI. Moreover, our results are similar to those found in studies in non-TAVI patients that did not show a beneficial effect of PCI on clinical outcomes [26, 27].

Interestingly, several studies included in our meta-analysis focused on patients undergoing PCI of left main or proximal segments—and observed similar mortality rates compared with patients treated with TAVI without PCI for these lesions [10, 17, 20]. These findings are of particular importance as current guidelines recommend revascularisation of these segments. Moreover, our sensitivity analyses on studies with a cut-off value of 50% for significant CAD showed a lower mortality risk in patients treated with TAVI only. PCI procedures in studies included in our sensitivity analysis were not guided by haemodynamic parameters, which may have resulted in revascularisation of intermediate lesions without haemodynamic significance. PCI in these patients could therefore have led to an unnecessary risk.

The conflicting results of previous studies on clinical outcomes in patients with significant CAD undergoing TAVI have led to extensive debate among cardiologists leading to varying strategies for the treatment of concomitant CAD in this patient population. Complete revascularisation of coronary arteries may lower the risk of MI during TAVI, specifically during hypotensive phases of the TAVI procedure (e.g. rapid pacing). Our study showed low and comparable incidences of MI in patients undergoing TAVI with and without preceding PCI, indicating that the choice to perform PCI did not have a significant effect on the occurrence of MI. Several underlying mechanisms for periprocedural MI have been described, including coronary embolisation by debris from the native aortic valve, coronary obstruction and severe hypotension [28]. Interestingly, none of the included studies reported on the type and subsequent treatment of MI in accordance with international guidelines. To better understand the occurrence of periprocedural MI in TAVI patients, future studies should report both MI type and treatment.

A second argument justifying PCI before TAVI is the coronary access after TAVI as the prosthesis may cause difficulties with cannulation of the coronary ostia. The risk of unsuccessful cannulation after TAVI may be increased by several factors, including the use of self-expandable valves. These technical challenges may lead to a greater risk of complications in patients undergoing PCI after TAVI. However, several observational studies have showed the feasibility of coronary angiography and subsequent PCI in TAVI patients. The largest study, including 15,000 TAVI patients, reported a low incidence of PCI after TAVI and a success rate of 97% without differences between types of TAVI prostheses [29]. Importantly, as TAVI indications expand towards younger patients, it is expected that the rate of PCI after TAVI will subsequently increase. More data on success rates of PCI, technical challenges and risk of complications are warranted.

A counterargument favouring a more conservative approach in patients with concomitant significant CAD undergoing TAVI is the risk of periprocedural complications during PCI. Life-threatening complications during PCI are less tolerated by patients with severe aortic valve stenosis due to their diminished ability to compensate for haemodynamic changes. Moreover, the use of DAPT after PCI results in an increased risk of bleeding during TAVI procedure. Our study showed an increased risk of major bleeding in patients with PCI prior to TAVI, reinforcing the argument for less aggressive therapy for significant CAD in the TAVI population as the rates of other clinical outcomes were comparable between the 2 treatment strategies. Interestingly, previous meta-analyses have not reported on bleeding complications despite their association with poor clinical outcomes [30]. Therefore, the need for PCI prior to TAVI should outweigh the bleeding risk in patients with coexisting aortic valve stenosis and significant CAD. In patients who are revascularised > 1 month before TAVI, shortening of the duration of DAPT may be a good strategy to mitigate the increased bleeding risk.

### Study limitations and future studies

Our study has several limitations. The most important limitation is the non-randomised design of most included studies, which was accompanied by low quality of evidence. The latter was primarily caused by a serious risk of bias due to the lack of standardised decision-making on which patient received PCI before TAVI and which patient did not. This may have led to differences in baseline characteristics. Additionally, only a minority of studies reported on the use of haemodynamic parameters and the severity and location of CAD. Specifically, information on the presence of CAD in SYNTAX segments 1, 5, 6 and 11 would have been of great value as current guidelines recommend revascularisation of these segments in patients planned to undergo TAVI. Furthermore, as mainly high-risk patients were assessed in the included studies, extrapolation to younger patients with lower risk profiles should be done with caution.

Moreover, PCI has a beneficial effect on patient-related outcomes in chronic coronary syndromes [26]. Future studies should not only assess hard clinical outcomes, but also symptom relief and quality of life. In that respect, information on the necessity of PCI in patients with persistent angina after TAVI should also be collected. Therefore, patient-related outcomes such as symptom relief and the need for PCI in patients with persistent angina after TAVI should be assessed in future studies. Lastly, the absence of definitions and adjudication of clinical endpoints using international guidelines contributed significantly to the low quality of evidence.

The aforementioned limitations show that well-organised RCTs are warranted. Both the Dutch PRO-TAVI (PeRcutaneous cOronary intervention before TAVI; ClinicalTrials.gov identifier: NCT05078619) and NOTION‑3 (Nordic Aortic Valve Intervention‑3; ClinicalTrials.gov identifier: NCT03058627) trials aim to elucidate the benefit of PCI in patients with untreated significant CAD undergoing TAVI. The results of these RCTs will help Heart Teams to decide on the optimal treatment for the individual patient with concomitant CAD scheduled for TAVI.

## Conclusion

This first systematic review and meta-analysis showed no differences in clinical outcomes between patients with concomitant significant CAD undergoing TAVI with and without PCI at different time intervals. However, patients with PCI did have a higher risk of major bleeding within 30 days after TAVI. Importantly, these results should be seen in the light of serious risk of bias in the included studies. Therefore, RCTs with a higher certainty of evidence are required to elucidate the necessity of PCI for concomitant significant CAD in patients scheduled to undergo TAVI.

### Supplementary Information

**Table S1** Search strategy

**Table S2** Events per study for short-term clinical outcomes

**Table S3** Events per study for mid-term clinical outcomes

**Table S4** Events per study for all-cause mortality > 1 year

**Table S5** Risk of bias in observational studies using GRADE approach

**Table S6** Risk of bias in randomised controlled trials using GRADE approach

**Table S7** Certainty of evidence

**Figure S1** Flowchart of systematic review of identified records

**Figure S2** Funnel plots for **a** all-cause mortality at 30 days and **b** all-cause mortality at 1 year

**Figure S3** Sensitivity analysis of studies defining significant CAD as stenosis ≥ 70%

**Figure S4 **Sensitivity analysis of studies defining significant CAD as stenosis ≥ 50%
