# Supplementary material for: Percutaneous coronary intervention in patients undergoing transcatheter aortic valve implantation: a systematic review and meta-analysis
Source: Neth Heart J. 2023 Nov 1;31(12):489–99. doi: 10.1007/s12471-023-01824-w (PMC10667197; doi:10.1007/s12471-023-01824-w)
Supplement: Supplementary file 1 — Table S1 Search strategy [file 12471_2023_1824_MOESM1_ESM.docx]

**Table S1** Search strategy

*PubMed*

tavi*[Title/Abstract] OR tavr*[Title/Abstract] OR Percutaneous aortic valve*[Title/Abstract] OR Transcatheter Aortic Valve implant*[Title/Abstract] OR transcatheter aortic valve replacement*[Title/Abstract] OR "Transcatheter Aortic Valve Replacement"[Mesh]

AND

PCI[Title/Abstract] OR percutaneous coronary*[Title/Abstract] OR coronary stenting[Title/Abstract] OR coronary revascularization[Title/Abstract] OR coronary revascularisation[Title/Abstract] OR coronary angioplasty[Title/Abstract] OR PTCA[Title/Abstract] OR percutaneous transluminal coronary angioplasty[Title/Abstract] OR percutaneous coronary intervention[MeSH]

*Embase*

('transcatheter aortic valve*':ti,ab,kw OR tavi:ti,ab,kw OR tavr:ti,ab,kw OR 'percutaneous aortic valve*':ti,ab,kw)

AND

('percutaneous coronary intervention*':ti,ab,kw OR 'coronary stenting':ti,ab,kw OR 'coronary angioplasty':ti,ab,kw OR 'percutaneous transluminal coronary angioplasty':ti,ab,kw OR pci:ti,ab,kw OR ptca:ti,ab,kw OR 'coronary revascularization':ti,ab,kw)

AND

[embase]/lim NOT [medline]/lim

*Cochrane*

(TAVI*):ti,ab,kw OR (TAVR*):ti,ab,kw OR (percutaneous aortic valve*):ti,ab,kw OR (transcatheter aortic valve implant*):ti,ab,kw OR (transcatheter aortic valve replacement*):ti,ab,kw

AND

(percutaneous coronary* OR coronary stenting OR PCI OR coronary revascularization OR coronary revascularization OR PTCA OR percutaneous transluminal coronary angioplasty OR coronary angioplasty):ti,ab,kw
