# Supplementary material for: Percutaneous coronary intervention in patients undergoing transcatheter aortic valve implantation: a systematic review and meta-analysis
Source: Neth Heart J. 2023 Nov 1;31(12):489–99. doi: 10.1007/s12471-023-01824-w (PMC10667197; doi:10.1007/s12471-023-01824-w)
Supplement: Supplementary file 2 — Table S2 Events per study for short-term clinical outcomes [file 12471_2023_1824_MOESM2_ESM.docx]

**Table S2** Events per study for short-term clinical outcomes

*All-cause mortality ≤ 30 days*

|  | **No PCI** | | **PCI** | |
| --- | --- | --- | --- | --- |
| **Study name** | **Events** | **No events** | **Events** | **No events** |
| Barbanti et al, 2017 | 1 | 82 | 1 | 50 |
| Zivelonghi et al, 2017 | 5 | 37 | 1 | 33 |
| Elyasi et al, 2018 | 3 | 70 | 2 | 90 |
| Huczek et al, 2018 | 27 | 266 | 13 | 156 |
| Caze et al, 2019 | 5 | 89 | 4 | 105 |
| Elbaz et al, 2020 | 32 | 412 | 29 | 415 |
| Young et al, 2020 | 0 | 58 | 6 | 96 |
| Dagan et al, 2021 | 2 | 87 | 2 | 46 |
| Duran Karaduman et al, 2021 | 7 | 55 | 2 | 63 |
| Kaihara et al, 2021 | 0 | 46 | 0 | 32 |
| Matta et al, 2021 | 4 | 113 | 7 | 248 |
| Patterson et al, 2021 | 6 | 110 | 3 | 116 |
| **Total** | 91 | 1420 | 72 | 1444 |

|  | **No PCI** | | **PCI** | |
| --- | --- | --- | --- | --- |
| **Study name** | **Events** | **No events** | **Events** | **No events** |
| Barbanti et al, 2017 | 0 | 83 | 1 | 50 |
| Zivelonghi et al, 2017 | 3 | 39 | 1 | 33 |
| Patterson et al, 2021 | 5 | 111 | 1 | 118 |
| **Total** | 8 | 233 | 3 | 201 |

*Cardiac death ≤ 30 days*

*Stroke ≤ 30 days*

|  | **No PCI** | | **PCI** | |
| --- | --- | --- | --- | --- |
| **Study name** | **Events** | **No events** | **Events** | **No events** |
| Barbanti et al, 2017 | 0 | 83 | 0 | 51 |
| Zivelonghi et al, 2017 | 0 | 42 | 0 | 34 |
| Young et al, 2020 | 0 | 58 | 6 | 96 |
| Duran Karaduman et al, 2021 | 0 | 62 | 0 | 65 |
| Kaihara et al, 2021 | 0 | 46 | 0 | 32 |
| Matta et al, 2021 | 3 | 114 | 6 | 249 |
| Patterson et al, 2021 | 4 | 112 | 5 | 114 |
| **Total** | 7 | 517 | 17 | 641 |

M*yocardial infarction ≤ 30 days*

|  | **No PCI** | | **PCI** | |
| --- | --- | --- | --- | --- |
| **Study name** | **Events** | **No events** | **Events** | **No events** |
| Zivelonghi et al, 2017 | 0 | 42 | 1 | 33 |
| Young et al, 2020 | 0 | 58 | 0 | 102 |
| Kaihara et al, 2021 | 1 | 45 | 0 | 32 |
| Patterson et al, 2021 | 2 | 114 | 5 | 114 |
| **Total** | 3 | 259 | 6 | 281 |

*Major bleeding ≤ 30 days*

|  | **No PCI** | | **PCI** | |
| --- | --- | --- | --- | --- |
| **Study name** | **Events** | **No events** | **Events** | **No events** |
| Barbanti et al, 2017 | 14 | 69 | 10 | 41 |
| Elbaz et al, 2020 | 24 | 420 | 34 | 410 |
| Duran Karaduman et al, 2021 | 0 | 62 | 1 | 64 |
| Matta et al, 2021 | 2 | 115 | 12 | 243 |
| Patterson et al, 2021 | 21 | 95 | 31 | 88 |
| **Total** | 61 | 761 | 88 | 846 |
