# Supplementary material for: Percutaneous coronary intervention in patients undergoing transcatheter aortic valve implantation: a systematic review and meta-analysis
Source: Neth Heart J. 2023 Nov 1;31(12):489–99. doi: 10.1007/s12471-023-01824-w (PMC10667197; doi:10.1007/s12471-023-01824-w)
Supplement: Supplementary file 3 — Table S3 Events per study for mid-term clinical outcomes [file 12471_2023_1824_MOESM3_ESM.docx]

**Table S3** Events per study for mid-term clinical outcomes

*All-cause mortality ≤ one year*

|  | **No PCI** | | **PCI** | |
| --- | --- | --- | --- | --- |
| **Study name** | **Events** | **No events** | **Events** | **No events** |
| Elyasi et al, 2018 | 16 | 73 | 17 | 92 |
| Caze et al, 2019 | 22 | 94 | 17 | 109 |
| Elbaz et al, 2020 | 75 | 444 | 79 | 444 |
| Boogert et al, 2021 | 37 | 427 | 28 | 150 |
| Dagan et al, 2021 | 6 | 89 | 4 | 48 |
| Duran Karaduman et al, 2021 | 11 | 62 | 9 | 65 |
| Kaihara et al, 2021 | 3 | 46 | 3 | 32 |
| Patterson et al, 2021 | 14 | 116 | 16 | 119 |
| **Total** | 182 | 1341 | 173 | 1053 |

*Cardiac death ≤ one year*

|  | **No PCI** | | **PCI** | |
| --- | --- | --- | --- | --- |
| **Study name** | **Events** | **No events** | **Events** | **No events** |
| Boogert at al, 2021 | 33 | 394 | 26 | 124 |
| Patterson et al, 2021 | 11 | 105 | 7 | 112 |
| **Total** | 44 | 499 | 33 | 236 |

*Myocardial infarction ≤ one year*

|  | **No PCI** | | **PCI** | |
| --- | --- | --- | --- | --- |
| **Study name** | **Events** | **No events** | **Events** | **No events** |
| Kaihara et al, 2021 | 3 | 43 | 1 | 31 |
| Patterson et al, 2021 | 4 | 112 | 8 | 111 |
| **Total** | 8 | 154 | 7 | 14 |
