# Supplementary material for: Percutaneous coronary intervention in patients undergoing transcatheter aortic valve implantation: a systematic review and meta-analysis
Source: Neth Heart J. 2023 Nov 1;31(12):489–99. doi: 10.1007/s12471-023-01824-w (PMC10667197; doi:10.1007/s12471-023-01824-w)
Supplement: Supplementary file 4 — Table S4 Events per study for all-cause mortality > 1 year [file 12471_2023_1824_MOESM4_ESM.docx]

**Table S4** Events per study for all-cause mortality > 1 year

|  | **No PCI** | | **PCI** | |
| --- | --- | --- | --- | --- |
| **Study name** | **Events** | **No events** | **Events** | **No events** |
| Milan-Iturbe et al, 2018 | 34 | 54 | 75 | 61 |
| Boogert et al, 2021 | 128 | 299 | 51 | 99 |
| **Total** | 162 | 353 | 126 | 160 |
