# Supplementary material for: Percutaneous coronary intervention in patients undergoing transcatheter aortic valve implantation: a systematic review and meta-analysis
Source: Neth Heart J. 2023 Nov 1;31(12):489–99. doi: 10.1007/s12471-023-01824-w (PMC10667197; doi:10.1007/s12471-023-01824-w)
Supplement: Supplementary file 5 — Table S5 Risk of bias in observational studies using GRADE approach [file 12471_2023_1824_MOESM5_ESM.docx]

**Table S5** Risk of bias in observational studies using GRADE approach

| **Study** | **Time interval** | **Standardized criteria for revascularization** | **Measurement of outcome** | **Confounders reported** | **Adjustment for confounding factors** | **Complete follow-up** |
| --- | --- | --- | --- | --- | --- | --- |
| Barbanti et al, 2017[12] | In-hospital | Standardized | In hospital; VARC-2 criteria | STS Score | No adjustment | 99% |
| Zivelonghi et al, 2017[8] | 30 days | Unstandardized | Outpatient clinic data or phone consultation; unknown criteria | Log EuroSCORE | No adjustment | Unknown |
| Elyasi et al, 2018[14] (Abstract) | 30 days  One year | Unknown | Unknown | None | N/A | Unknown |
| Huczek et al, 2018[19] | 30 days | Unstandardized | Ambulatory visits and civil registry; unknown criteria | Syntax Score | No adjustment | Unknown |
| Millan-Iturbe et al, 2018[15] | Nine years | Unstandardized | Civil registry; unknown criteria | EuroSCORE  STS score | No adjustment | 99% |
| Cazé et al, 2019[18] (Abstract) | 30 days  One year | Unknown | Unknown | Syntax Score | Similar between groups | Unknown |
| Elbaz et al, 2020[21] | In-hospital  30 days  One year | Unknown | Civil registry | None | N/A | Unknown |
| Young et al, 2020[17] (Abstract) | In-hospital  30 days | Unknown | In hospital, 30-day unknown; unknown criteria | None | N/A | Unknown |
| Boogert et al, 2021[20] | Six months  Three years | Unstandardized | Civil registry; VARC-2 criteria | EuroSCORE II  STS Score | No adjustment | 100% |
| Dagan et al, 2021[13] | 30 days  One year | Unknown | Follow-up visits; unknown criteria | STS PROM | No adjustment | Unknown |

| Duran Karaduman et al, 2021[11] | In-hospital  30 days  Six months  One year | Unstandardized | Unknown; VARC-2 criteria | Log EuroSCORE  EuroSCORE II  STS score  Syntax score | Similar between groups | Unknown |
| --- | --- | --- | --- | --- | --- | --- |
| Kaihara et al, 2021[10] | Two year | Unstandardized | Clinical records; unclear criteria | Log EuroSCORE  STS Score  Syntax score | Yes, STS score | Unknown |
| Matta et al, 2021[9] | In-hospital | Unstandardized | In-hospital; VARC-2 | EuroSCORE  STS-PROM | Unclear | Unknown |

Abbreviations: EuroSCORE, the European System for Cardiac Operative Risk Evaluation; STS-PROM, Society of Thoracic Surgeons Predicted Risk of Mortality; STS, Society of Thoracic Surgeons score; VARC, Valve Academic Research Consortium.
