# Supplementary material for: Percutaneous coronary intervention in patients undergoing transcatheter aortic valve implantation: a systematic review and meta-analysis
Source: Neth Heart J. 2023 Nov 1;31(12):489–99. doi: 10.1007/s12471-023-01824-w (PMC10667197; doi:10.1007/s12471-023-01824-w)
Supplement: Supplementary file 6 — Table S6 Risk of bias in randomised controlled trials using GRADE approach [file 12471_2023_1824_MOESM6_ESM.docx]

**Table S6** Risk of bias in randomised controlled trials using GRADE approach

| **Study** | **Time interval** | **Allocation concealment** | **Blinding** | **Follow-up and outcome events** | **Reporting bias** | **Other limitations** |
| --- | --- | --- | --- | --- | --- | --- |
| Patterson et al, 2021[16] | 30 days  One year | Electronic system used, varying block sizes | Open-label | 10% was lost during follow-up, both intention-to-treat and as-treated analyses were performed | No | No |
