# Supplementary figures and images for: Percutaneous coronary intervention in patients undergoing transcatheter aortic valve implantation: a systematic review and meta-analysis
Source: Neth Heart J. 2023 Nov 1;31(12):489–99. doi: 10.1007/s12471-023-01824-w (PMC10667197; doi:10.1007/s12471-023-01824-w)

**
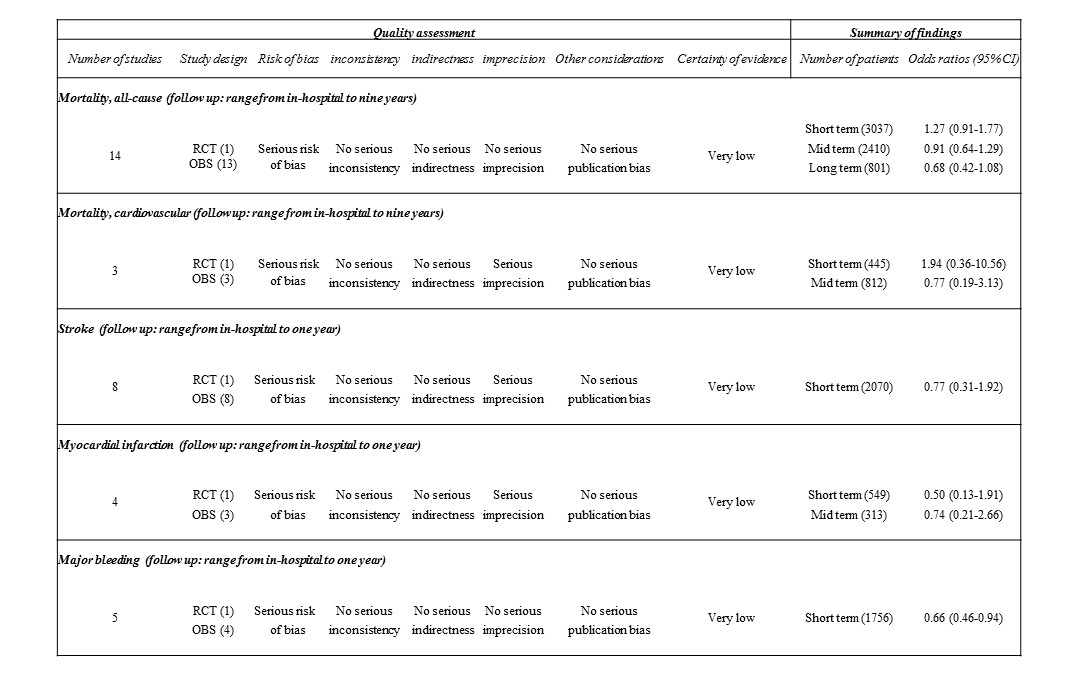
Table S7** Certainty of evidence

Supplement: Supplementary file 7 — Table S7 Certainty of evidence [file 12471_2023_1824_MOESM7_ESM.docx]
