# Supplementary material for: Percutaneous coronary intervention in patients undergoing transcatheter aortic valve implantation: a systematic review and meta-analysis
Source: Neth Heart J. 2023 Nov 1;31(12):489–99. doi: 10.1007/s12471-023-01824-w (PMC10667197; doi:10.1007/s12471-023-01824-w)
Supplement: Supplementary file 8 — Figure S1 Flowchart of systematic review of identified records [file 12471_2023_1824_MOESM8_ESM.docx]

**Figure S1** Flowchart of systematic review of identified records. *CAD* coronary artery disease

**Records identified
(n = 1640)**

**Screening**

**Included**

**Eligibility**

**Identification**

**Records screened
(n = 1443)**

**Records excluded
(n = 1357)**

Not relevant n = 1314

No original data n = 39

Ongoing trial n = 4

**Full-text articles assessed for eligibility
(n = 86)**

**Studies excluded**

**(n = 72)**

No control group or absence of concomitant CAD n = 64

No data reported n = 6

Similar data n = 2

**Studies included in qualitative synthesis
(n = 14)**

**Duplicates removed**

**(n = 197)**
