# Supplementary material for: Percutaneous coronary intervention in patients undergoing transcatheter aortic valve implantation: a systematic review and meta-analysis
Source: Neth Heart J. 2023 Nov 1;31(12):489–99. doi: 10.1007/s12471-023-01824-w (PMC10667197; doi:10.1007/s12471-023-01824-w)
Supplement: Supplementary file 9 — Figure S2 Funnel plots for a all-cause mortality at 30 days and b all-cause mortality at 1 year [file 12471_2023_1824_MOESM9_ESM.docx]

**Figure S2** Funnel plots for **a** all-cause mortality at 30 days and **b** all-cause mortality at 1 year. Both raised concern for publication bias. Egger test did not confirm (*p*> 0.05)

**
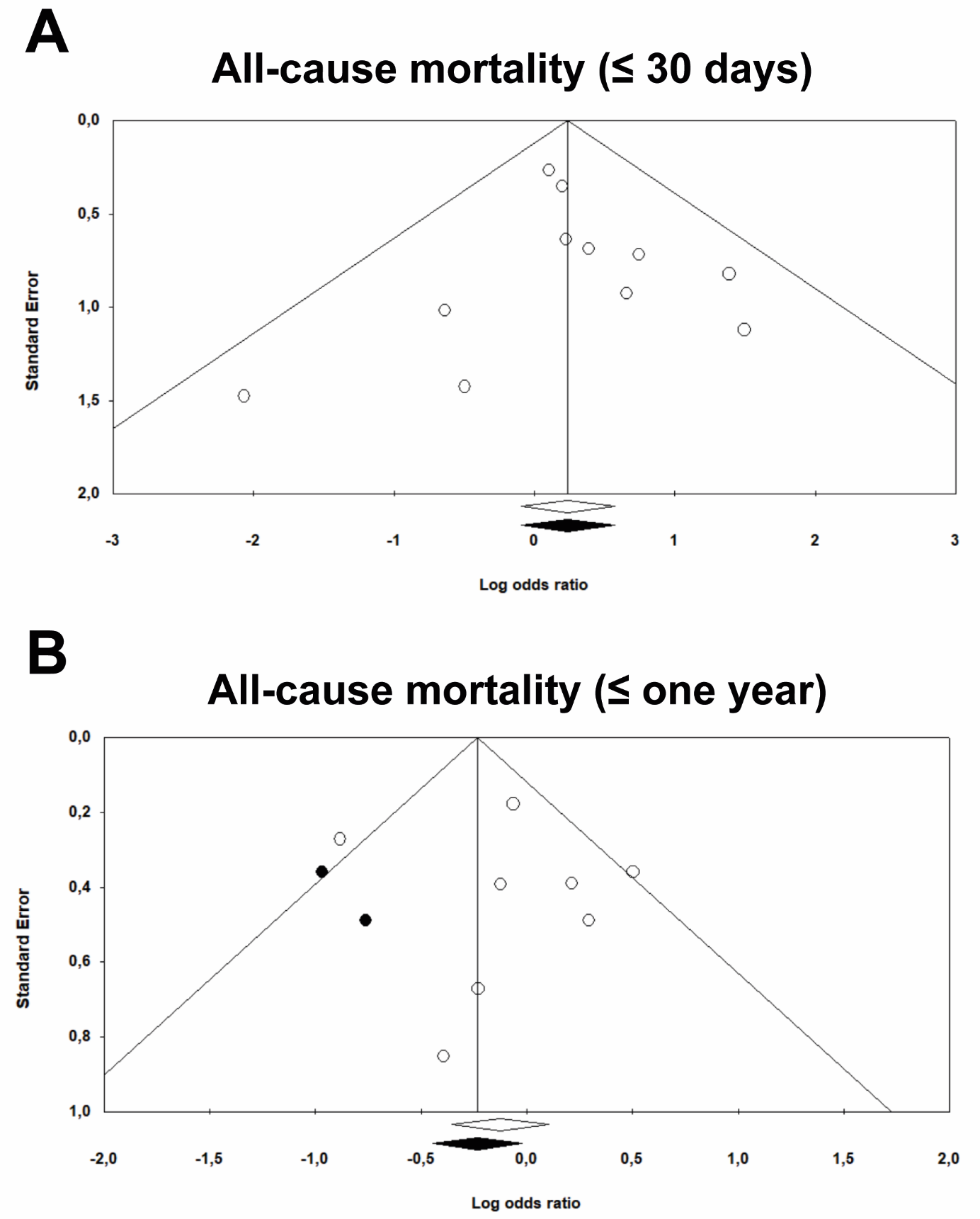
**
