# Supplementary material for: Percutaneous coronary intervention in patients undergoing transcatheter aortic valve implantation: a systematic review and meta-analysis
Source: Neth Heart J. 2023 Nov 1;31(12):489–99. doi: 10.1007/s12471-023-01824-w (PMC10667197; doi:10.1007/s12471-023-01824-w)

**Figure S3** Sensitivity analysis of studies defining significant CAD as stenosis ≥ 70%. Funnel plots for (A) all-cause mortality at 30 days, (B) cardiac death at 30 days, (C) stroke at 30 days, (D) myocardial infarction at 30 days, (E) major bleeding at 30 days, (F) all-cause mortality at 1 year, and (G) myocardial infarction at 1 year. *PCI* percutaneous coronary intervention


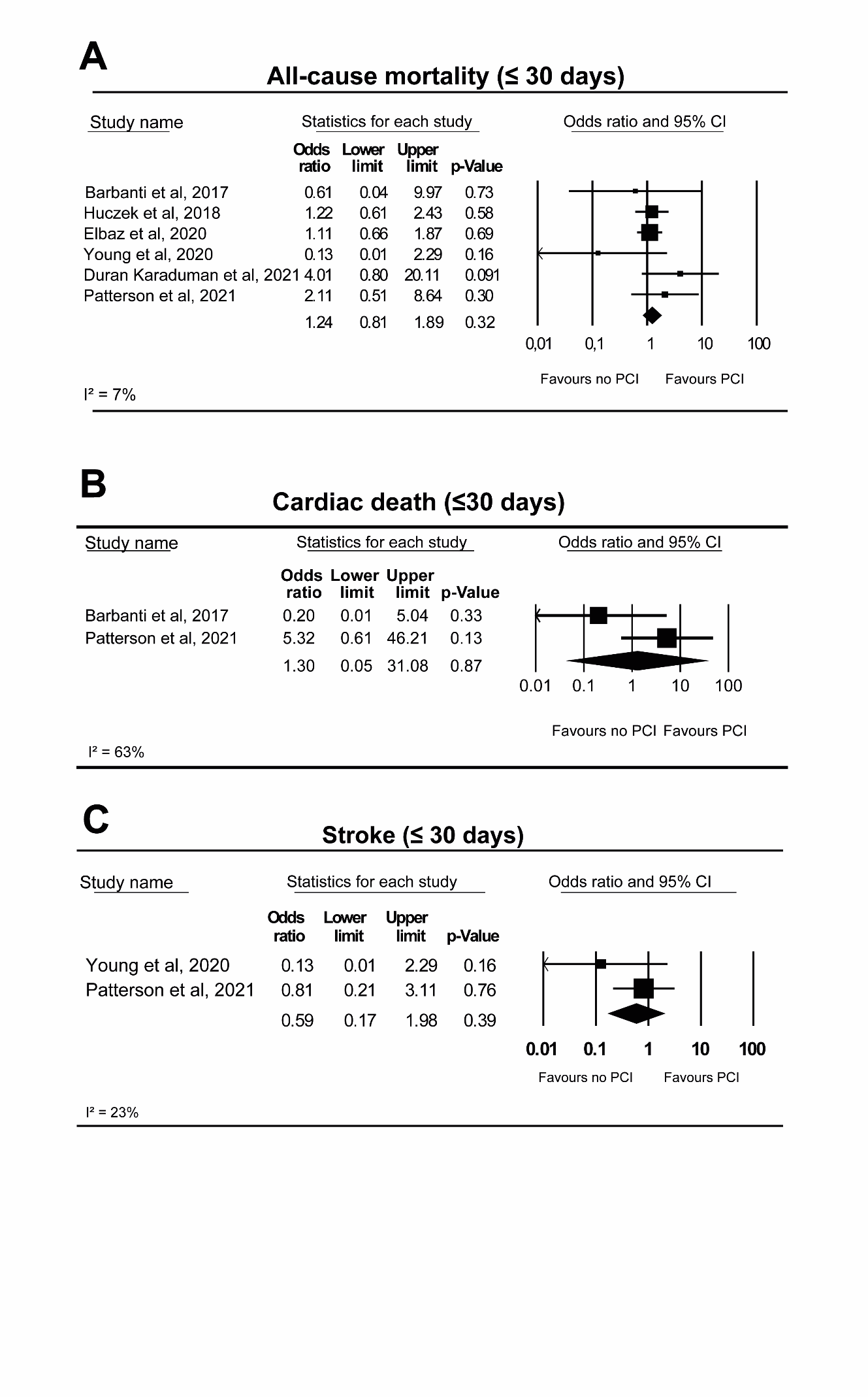


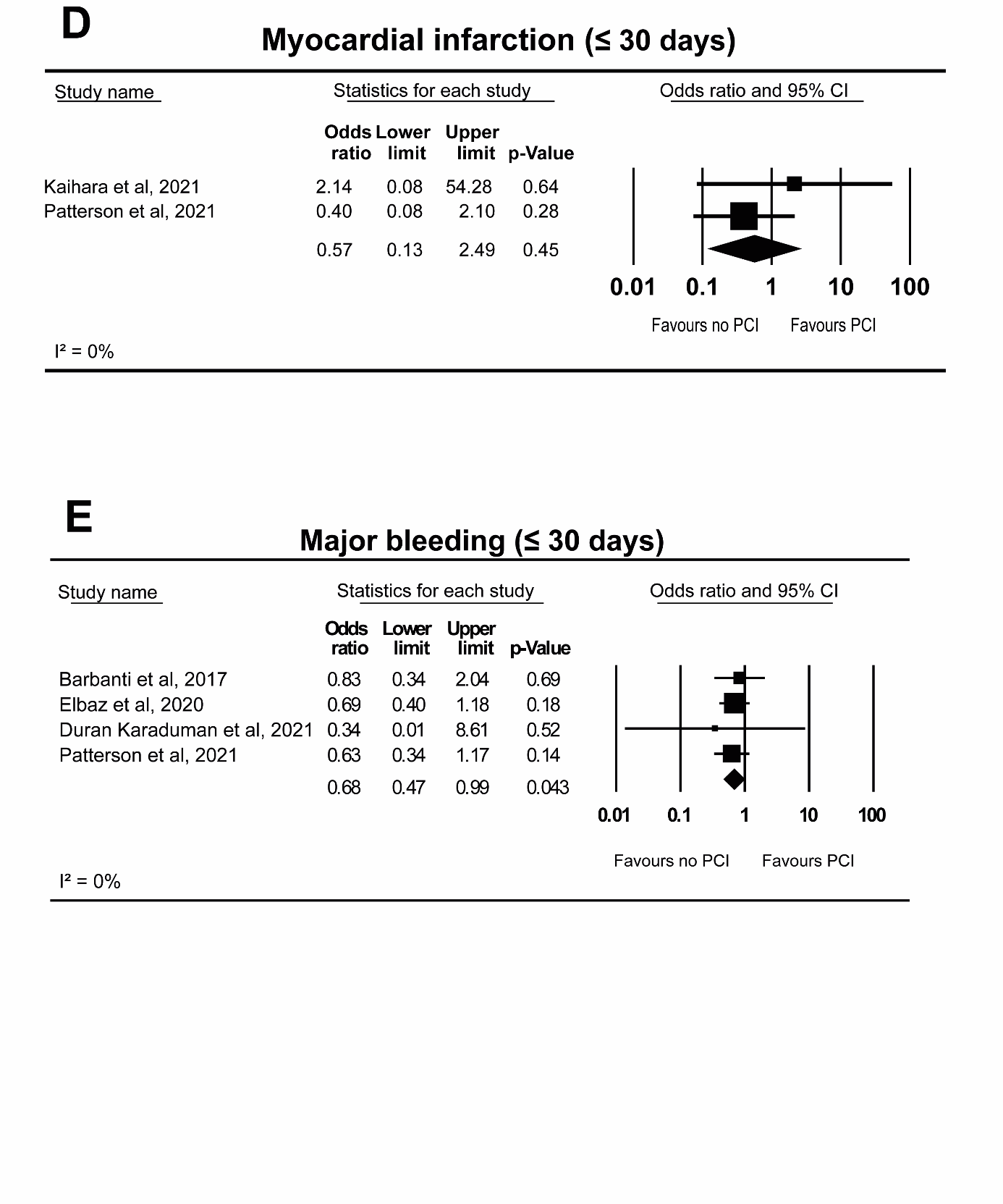


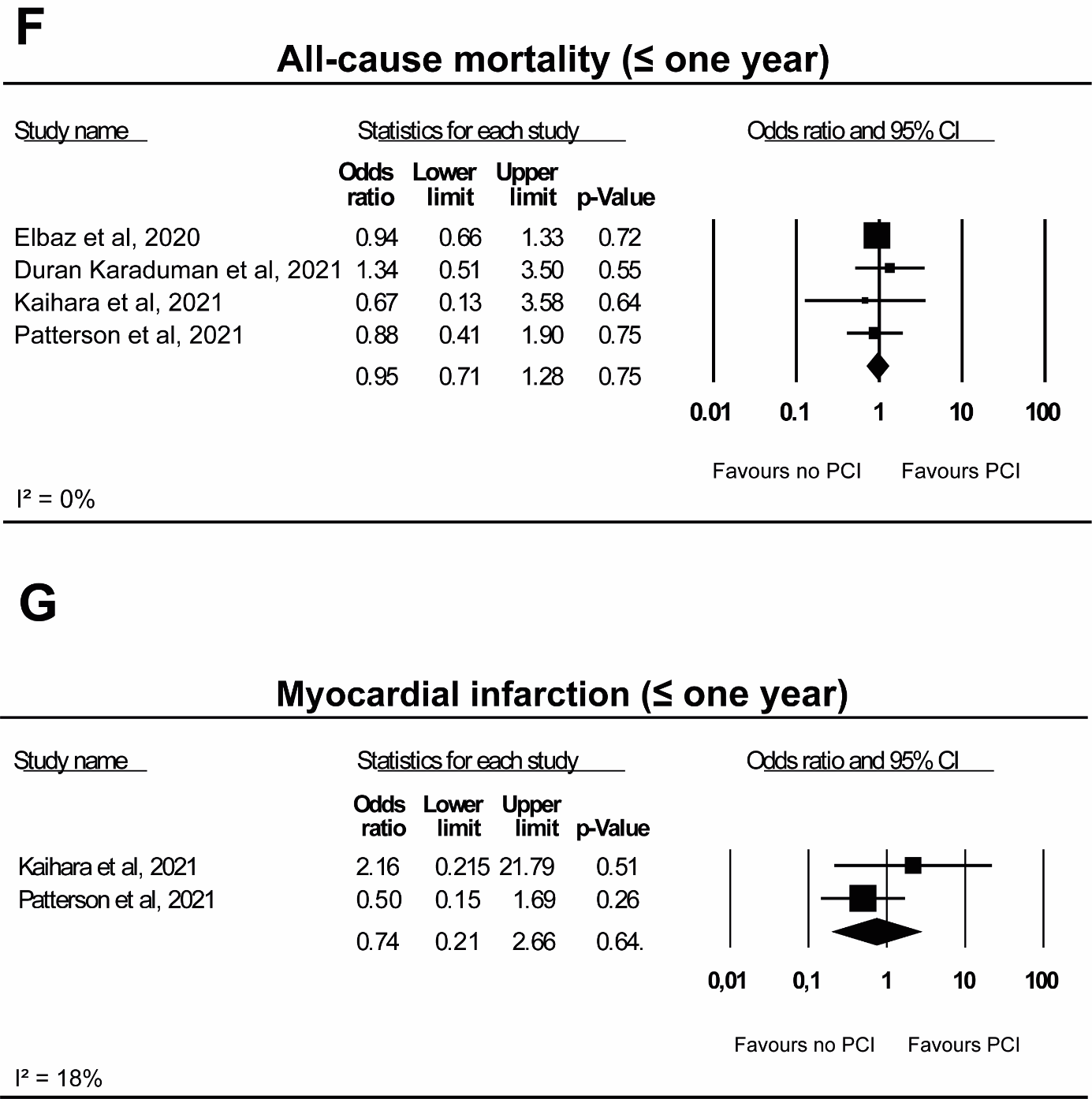

Supplement: Supplementary file 10 — Figure S3 Sensitivity analysis of studies defining significant CAD as stenosis ≥ 70% [file 12471_2023_1824_MOESM10_ESM.docx]
