# Supplementary material for: Percutaneous coronary intervention in patients undergoing transcatheter aortic valve implantation: a systematic review and meta-analysis
Source: Neth Heart J. 2023 Nov 1;31(12):489–99. doi: 10.1007/s12471-023-01824-w (PMC10667197; doi:10.1007/s12471-023-01824-w)

**Figure S4** Sensitivity analysis of studies defining significant CAD as stenosis ≥ 50%. Funnel plots for (A) all-cause mortality at 30 days, and (B) all-cause mortality at 1 year. PCI percutaneous coronary intervention


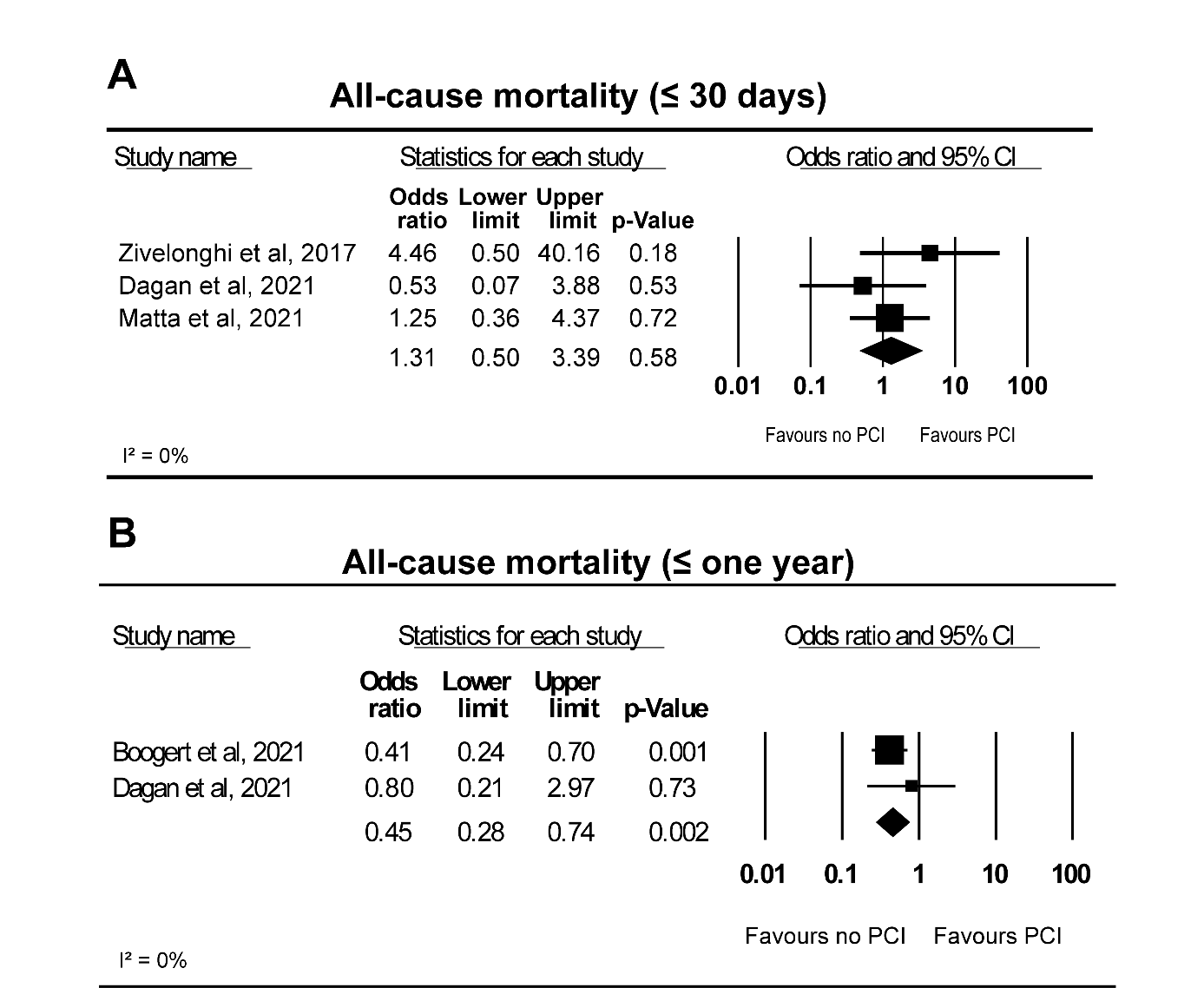

Supplement: Supplementary file 11 — Figure S4 Sensitivity analysis of studies defining significant CAD as stenosis ≥ 50% [file 12471_2023_1824_MOESM11_ESM.docx]
